# Measurement invariance of cognitive and affective job insecurity: A cross-national study

**DOI:** 10.4102/ajopa.v6i0.147

**Published:** 2024-04-25

**Authors:** Gina Görgens-Ekermans, Valerio Ghezzi, Tahira M. Probst, Claudio Barbaranelli, Laura Petitta, Lixin Jiang, Sanman Hu

**Affiliations:** 1 Department of Industrial Psychology, Faculty of Economic and Management Sciences, Stellenbosch University, Cape Town, South Africa; 2 Department of Psychology, Sapienza University of Rome, Rome, Italy; 3 Department of Psychology, College of Arts and Sciences, Washington State University, Vancouver, United States of America; 4 School of Psychology, University of Auckland, Auckland, New Zealand; 5 School of Business, Huaqiao University, Quanzhou, China

**Keywords:** bifactor-(S−1) model, cross-national comparison, cognitive job insecurity, affective job insecurity, measurement invariance

## Abstract

Empirical evidence of established measurement invariance of job insecurity measures may enhance the practical utility of job insecurity as a valid predictor when utilised over different cross-national samples. This study investigated the measurement invariance of the nine-item versions of the Job Security Index (a measure of cognitive job insecurity) and the Job Security Satisfaction Scale (a measure of affective job insecurity), across four countries (i.e. the United States, *N* = 486; China, *N* = 629; Italy, *N* = 482 and South Africa, *N* = 345). Based on a novel bifactor-(S-1) model approach we found evidence for partial metric, partial scalar and partial strict invariance of our substantive bifactor-(S-1) structure. The results extend measurement invariance research on job insecurity with obvious pragmatic implications (e.g. scaling units, measurement bias over cross-national interpretations).

**Contribution:**

This research provides evidence to support the applied use of cross-national comparisons of job insecurity scores across the nationalities included in this study. Theoretically, this research advances the debate about the nature of the relationship between cognitive and affective job insecurity, suggesting that in this cross-national dataset, a model where cognitive job insecurity is specified as the reference domain outperforms a model where affective job insecurity is assigned this status. Practically, it demonstrates that it is sensible and necessary to differentiate between cognitive and affective job insecurity and include measures of both constructs in future research on the construct.

## Introduction

Globalisation, technological advances and changing government policies regarding work and labour relations have led to an increase in organisational restructuring, downsizing and mergers (Hirsch & De Soucey, 2006; László et al., 2010). At the same time, the nature of work arrangements has shifted away from permanent full-time positions towards an increasing reliance on precarious employment relationships in a so-called gig economy (e.g. part-time, contingent and/or independent work contracts) (MacDonald & Giazitzoglu, 2019). For example, in 2018, it was estimated that informal work accounts for roughly 61% of the global workforce (ILO, 2018). A 2014 survey of corporate executives in 27 countries (Oxford Economics, 2014) found that a large majority (83%) intended to increase their reliance on contingent, intermittent and contract employees. Indeed, non-standard precarious work arrangements accounted for 60% of all new jobs created in countries, listed as part of the Organisation for Economic Cooperation and Development (OCED), from 2007 to 2013 and one-third of all jobs total (Kalleberg, 2018). While the gig economy and non-standard work arrangements might allow greater flexibility in where, when and how work is completed, collectively these global trends compounded by intermittent economic downturns and financial crises have led to pervasive job insecurity among today’s workers worldwide (Probst et al., 2023).

In addition, the outbreak of the global coronavirus disease 2019 (COVID-19) pandemic caused a historic number of job losses worldwide (e.g. Blustein et al., 2020), with some regions being more affected than others (i.e. higher in America, compared to Europe and central Asia; Eurofound, 2021), and unemployment trends surpassing the 2008–2009 financial crisis (i.e. global labour force participation rates down by 2.2%, compared to 0.2%, ILO, 2021a). More specifically, in January 2021, the International Labour Organization (ILO) estimated a global loss of 114 million jobs (relative to 2019), reflecting the 2020 impact of the pandemic (ILO, 2021a), a trend which worsened into 2021 with 137 million job losses reflected in the third quarter of 2021 (ILO, 2021b). Moreover, pre-pandemic trends (e.g. ILO, 2013) of a reduction of youth employment opportunities, coupled with a narrower selection of types of jobs, employment opportunities and job conditions, as a result of the ongoing financial crisis, especially in Europe, were exacerbated by the pandemic. These fundamental changes in the nature of work coupled with economic and financial crises have led some researchers (e.g. Jiang & Probst, 2014; Kalleberg, 2013) to argue that job insecurity is a critical and ubiquitous stressor in today’s workplaces.

Nearly four decades of research on job insecurity has provided a strong body of evidence underscoring the numerous adverse consequences associated with this workplace stressor. A comprehensive meta-analysis (Jiang & Lavaysse, 2018) summarising that the body of literature encompassed 535 independent samples with sample sizes up to *N* = 300 000 employees. The meta-analytic results provided compelling evidence of adverse effects of job insecurity on a wide range of job-related (e.g. commitment, absenteeism, safety behaviours, accidents, motivation and citizenship behaviours) and individual outcomes (e.g. physical and mental health). Moreover, a 2019 meta-analysis by Sverke et al. (2019) on 119 samples covering 106 studies, provided strong evidence of the pervasive effect of job insecurity on a range of job performance outcomes (e.g. task performance, counterproductive work behaviour and contextual performance).

These adverse effects of job insecurity have been observed in research conducted around the world and in numerous different settings. For example, two large-scale European studies (László et al., 2010; Probst & Jiang, 2017) on pooled data from 19 and 16 European countries, respectively, investigated job insecurity in relation to health and job stress. Many comprehensive studies on United States (US) workers (e.g. Jiang & Probst, 2014; Lawrence & Kacmar, 2017; Probst et al., 2021) exist. In the last 7 years, research on German (e.g. Barrech et al., 2018; Helbling & Kanji, 2018), Swedish (Låstad et al., 2018), Canadian (Watson & Osberg, 2018), Chinese (Lin et al., 2021; Wang et al., 2018), Italian (Chirumbolo et al., 2021; Probst et al., 2018), Taiwanese (Hsieh & Huange, 2018), Turkish (Bitmiş & Ergeneli, 2015), Flemish (Griep et al., 2021), Swiss (Sender et al., 2016) and South African (e.g. De Beer et al., 2016; Smit et al., 2016) employees have been published. The global reach of research efforts on job insecurity is further evidenced in a very recent meta-analysis by Jiang et al. (2021) encompassing 429 samples from 39 countries which investigated the sources of job insecurity through a resources-demands perspective.

Clearly, job insecurity is a pervasive workplace stressor with numerous adverse outcomes observed across different national and cultural settings. Yet, despite the clear relevance of job insecurity globally, there have been surprisingly few attempts to determine whether the measurement of the job insecurity construct is invariant across those different settings. Importantly, when measurement instruments are transported from one country to another, the comparability of those psychological measurements across different groups should be investigated. More specifically, tests of bias and equivalence should routinely be conducted so that bias and equivalence investigations could have theoretical and practical relevance. The presence of construct, method or item bias could express itself in the structural, metric and/or scalar non-equivalence of the given instruments.

Comporting with Cheung and Rensvold’s (2002) admonition regarding the importance of assessing measurement equivalence, we argue that invariance testing of job insecurity measures should be assessed before attempting to compare or interpret mean differences on the latent trait across groups. If measurement invariance or equivalence assumptions remain untested, the practical utility of job insecurity as a valid predictor when utilised over different cross-national groups may be questionable. Therefore, the purpose of the current study was to evaluate the measurement invariance across four countries (China, Italy, South Africa and the US) of two widely used measures of job insecurity (the Job Security Index, JSI; and the Job Security Satisfaction scale, JSS) initially developed by Probst (2003) for use in the United States with an English-speaking population. Our results could help inform future comparative international research and facilitate accurate interpretation of cross-country comparisons of the job security construct when measured with these scales. Below, we begin by discussing historical and more recent conceptual definitions and operationalisations of the job insecurity construct, with a focus on the measures being tested in the current study. Next, we summarise cross-cultural and cross-national studies of job insecurity, while noting the few instances where measurement equivalence has been explicitly assessed. Finally, we present the measurement invariance hypotheses being tested in the current study.

### Conceptualisations and operationalisations of the job insecurity construct

The absence of a coherent conceptual definition and operationalisation of the job insecurity construct has plagued research endeavours in this field since the early seminal studies (Probst, 2003). Specifically, researchers have long-debated: (1) whether job insecurity should be conceptualised and measured as a subjective experience (i.e. something that is in the eye of the beholder) or an objective state (e.g. threatened by a layoff or a contingent employment status); (2) whether job insecurity is best conceptualised as a unidimensional vs. multidimensional construct and (3) whether it is theoretically or practically meaningful to differentiate between cognitive and affective insecurity.

While some disciplines (e.g. economics) prefer to conceptualise job insecurity as an objective state, researchers within the field of psychology have traditionally viewed it as best understood and described as a subjective phenomenon (e.g. Greenhalgh & Rosenblatt, 1984; Probst, 2002; Sverke, Hellgren, & Naswall, 2002). Using this perspective, job insecurity has been defined (and will be defined in the current study) as *the subjective perception that the future of one’s job is unstable or at risk*, regardless of actual objective levels of job security (Probst et al., 2018).

Researchers next grappled with whether to view the construct as unidimensional versus multidimensional. Unidimensional definitions and measures of job insecurity tend to take a global approach encompassing the perceived job security associated with the totality of one’s job (e.g. expectations of a change in their job for the worse). On the other hand, multidimensional measures (e.g. Hellgren et al., 1999) explicitly differentiate between quantitative job insecurity (i.e. threats of job loss) and qualitative job insecurity (i.e. threats to valued features of one’s job). While there are pros and cons to each approach, Reisel and Banai (2002) found that the threat of job loss was a better predictor of employee outcomes than a threat to their job features; moreover, a global (unidimensional) measure of job insecurity generally explained more variance than a multidimensional measure. Thus, in the current study, we focused on unidimensional global measures of job insecurity, specifically one that assesses cognitive job insecurity and the other measuring affective job insecurity.

Since the initial distinction between cognitive and affective job insecurity proposed by Borg and Elizur (1992), research has indeed increasingly suggested that there is value in this approach (Huang et al., 2010; Jiang & Probst, 2014; Jiang et al., 2020; Probst, 2003; Reisel & Banai, 2002). Whereas cognitive job insecurity reflects an employee’s appraisal of the future (in)stability of his or her job, affective job insecurity reflects employee affective reactions regarding those perceived levels of job insecurity (Probst, 2003). Thus, cognitive job insecurity reflects perceptions regarding the *likelihood* of negative changes to one’s job (e.g. with respect to job loss or loss of valued job features), whereas affective job insecurity refers to *emotional reactions* to that potential loss (e.g. concern, worry and anxiety). Empirical evidence suggests that cognitive and affective job insecurity have differential relationships with antecedents and consequences and supports the validity of making this distinction (Bazzoli & Probst, 2023; Huang et al., 2010; Jiang & Lavaysse, 2018; Jiang et al., 2021; Probst, 2003).

To address these issues, Probst (2003) developed and validated the Job Security Index (JSI) and Job Security Satisfaction (JSS) scale. Both measures assume that job insecurity is a subjective phenomenon and best measured using a global unidimensional approach (i.e. encompassing the entirety of the job, rather than job loss vs. loss of certain job features). However, the scales differentiate between the cognitive and affective aspects of the construct. Whereas the Job Security Index assesses ‘an individual’s cognitive appraisal of the future of his or her job with respect to the perceived level of stability and continuance of that job’ (Probst, 2003, p. 452), the Job Security Satisfaction scale was developed to assess ‘an individual’s attitudes regarding that level of job security (i.e. their affective reactions)’ (Probst, 2003, p. 452). This distinction between cognitive and affective job insecurity has increasingly been adopted by researchers in this domain (Jiang & Probst, 2014). Since their development and validation, the JSI and JSS scales have been used in more than 40 studies to date and in countries as varied as China, Nigeria, the US, Italy, Chile and Turkey.

Despite the extent to which these scales have been used, there has been limited empirical assessment of the measurement invariance of the scales across different cultural settings. However, as we will review below, this is not a unique shortcoming specific to these particular scales, but rather descriptive of much of the cross-cultural and cross-national studies on job insecurity (regardless of the measures used).

### Cross-cultural and cross-national studies on job security

In a meta-analysis of job insecurity, Jiang and Lavaysse (2018) identified 435 published articles with 535 independent samples. A number of these studies investigated aspects of the job insecurity construct across different language, culture and/or national contexts. For example, Roll et al. (2015) investigated the relationship between job insecurity and performance across two cross-national samples (i.e. Germany and China), while König et al. (2011) conducted a Swiss–US comparison of the correlates of job insecurity. Earlier studies include a cross-national study on job insecurity conducted on data from Israel, the Netherlands and the United Kingdom (UK) (van Vuuren et al., 1991), while a 30-country study on its relationship with high involvement work systems was conducted by Bacon and Blyton (2001). In 1993, Orpen published differential correlations on the relationship between job insecurity and psychological well-being among black and white South African employees (Orpen, 1993). Lee et al. (2008) developed a measure of job insecurity and validated it on data from China and the US.

### Assessing measurement invariance across groups

Only a few of the studies listed above conducted rigorous invariance or equivalence tests on the respective instruments utilised in their studies. For example, König et al. (2011) conducted a composite assessment (i.e. tested one measurement model) of the measurement equivalence of the job insecurity, job satisfaction, organisational commitment, turnover intention and uncertainty avoidance scales included in their study. Item parcels were constructed, and three models were tested (unrestricted mean and factor loadings, restricted factor loadings, and restricted mean and factor loadings). Configural but neither metric nor scalar invariances were obtained. Unfortunately, because the authors only tested the measurement equivalence of all scales used in their study, it is unknown whether the non-invariance was a result of the job insecurity measures. In addition, in the study by Lee et al. (2008) on the development of a cross-culturally appropriate measure of job insecurity, only individual country CFA results of the job insecurity measure in the two separate US and Chinese samples were reported. Because no further measurement invariance or equivalence tests across the groups were conducted, it is not known whether that measure of job insecurity functioned the same across the two groups.

In a South African study conducted by Pienaar et al. (2013), sufficient factor structure equivalence (by calculating Tucker’s Φ coefficient of congruence, see Van de Vijver & Leung, 1997) of a shortened version of the De Witte (2000) job insecurity measure was reported between black and white respondents. Further, more stringent invariance tests were also conducted by testing for factor loading and intercept invariance. The authors report support for weak factorial invariance by race (ΔCFI = 0.007) and conclude that when the intercepts were also constrained across groups, ‘total changes in CFI were slightly above the cut-off value of 0.01 for race (ΔCFI = 0.017)’ (Pienaar et al., 2013, p. 13).

Finally, Vander Elst et al. (2014) conducted a series of measurement invariance tests on the four-item JIS developed by De Witte (2000). Data derived from the translated versions were obtained from five West European countries and languages (i.e. Belgium [Flemish], The Netherlands [Dutch], Spain [Spanish], Sweden [Swedish] and the UK [English]). The results revealed evidence of full configural and metric invariance, as well as partial scalar and error variance invariance. Full factor variance invariance was also evident. The authors concluded that construct validity of the different translations of the JIS exists (Vander Elst et al., 2014). More recently, Shoss et al. (2023) reported metric invariance of the JIS (as part of the larger measurement model) over data from the US, UK and Belgium.

While the Vander Elst et al. (2014) study represents perhaps the most rigorous test of invariance to date, the De Witte JIS differs from the scales examined in the current study in that the De Witte JIS is a global unidimensional measure that contains items reflecting both cognitive and affective insecurity. On the other hand, the Probst (2003) Job Security Index and Job Security Satisfaction measures respond to calls and empirical evidence suggesting that these two forms of job insecurity reflect different unique constructs and should be measured and modelled as such (Jiang & Lavaysse, 2018).

### The present study

We argue that invariance research (at different levels) is needed to validate the cross-national use of job insecurity measures. Employing psychological measures in distinct contexts (e.g. cultural or language groups) requires the separation of cultural bias (e.g. construct, method, item bias; Van de Vijver & Tanzer, 2004) from true construct variance in the data attained from such measures, as observed group differences may be as a result of measurement bias and not real underlying differences (Cheung & Rensvold, 2002). This may impede consistent and reliable cross-study and cross-country comparisons (e.g. Vander Elst et al., 2014). To this end, this study aims to add to the scant body of knowledge of cross-cultural invariance analysis on two well-validated and widely used job insecurity measures by examining the configural, metric, scalar and strict invariance (e.g. Vandenberg & Lance, 2000) of the Job Security Index and the Job Security Satisfaction scales (JSI and JSS; Probst, 2003) measurement models over four cross-national samples.

## Method

### Study design

A quantitative, cross-sectional research design was employed in this research. Data collection took place from 2015 to 2019 (i.e. October 2017 – November 2017 in Italy, July 2017 – August 2017 in China, May 2015 in the US, and June 2019 – July 2019 in SA). All the datasets (China: *N* = 629; Italy: *N* = 482; South Africa [SA]: *N* = 345; US: *N* = 486) contained anonymous data.

### Participants

A description of the different subsamples in terms of available matched demographic information (only age and gender were available in the SA sample) is contained in Table 1. Table S1 of Online Appendix 1 reports frequencies regarding the employee distribution across industry sectors for the Chinese, Italian and US samples. As can be noted, the SA and Italian samples were (on average) slightly older than the others, while all samples were fairly balanced in terms of gender composition. With regards to Chinese, Italian and US sample comparisons, the Chinese sample displayed (on average) a higher number of years of education than the others, while the proportion of US employees unemployed during the past 5 years was slightly higher than for the Chinese and the Italian samples. In terms of job contracts and job types, we observed a significantly lower proportion of employees with permanent arrangements for the Chinese sample and a higher proportion of part-time employees for the Italian sample than for the others. Finally, both Chinese and US employees reported a higher number of working hours in a typical week than the Italian sample.

**TABLE 1:** Sub-sample characteristics and cross-cultural comparisons.

Characteristic	Sub-samples				Test statistic	Effect size *T*
	Chinese (*N* = 629)	Italian (*N* = 482)	SA (*N* = 345)	US (*N* = 486)		
**Age (M; s.d.)**	35.72^a^; 9.73	43.80^b^; 13.24	44.58^b^; 10.85	35.22^a^; 11.53	F_(3,1930)_ = 94.25***	Partial η^2^ = 0.128
**Gender**	-	-	-	-	$\chi_{\left(3\right)}^{2}$ = 10.09*	Cramer V = 0.075
Male (%)	47.90	51.70	46.1	55.70	-	-
Female (%)	52.10	48.30	53.9	44.30	-	-
**Years of education (M; s.d.)**	15.55^b^; 2.3	14.15^a^; 6.1	-	14.57^a^; 3.48	F_(2,1570)_ = 35.10***	Partial η^2^ = 0.043
**Marital status**	-	-	-	-	$\chi_{\left(6\right)}^{2}$ = 186.54***	Cramer V = 0.242
Unmarried (%)	15.70	32.10	-	42.50	-	-
Married/living together (%)	81.10	53.60	-	48.00	-	-
Separated/divorced (%)	2.90	11.80	-	8.50	-	-
Widowed (%)	0.30	2.50	-	1.0	-	-
**Including yourself, how many people live in your household? (M; s.d.)**	3.41^c^; 1.09	3.03^b^; 1.23	-	2.68^a^; 1.43	F_(2,1577)_ = 54.17***	Partial η^2^ = 0.064
**Unemployed during the past five years?**	-	-	-	-	$\chi_{\left(2\right)}^{2}$ = 83.87***	Cramer V = 0.230
Yes (%)	6.60	18.00	-	24.70	-	-
No (%)	93.40	82.00	-	75.30	-	-
**Job tenure (M; s.d.)**	6.61^b^; 7.18	12.28^c^; 10.88	-	4.19^a^; 3.18	F_(2,1577)_ = 116.95***	Partial η^2^ = 0.130
**Contract**	-	-	-	-	$\chi_{\left(4\right)}^{2}$ = 308.36***	Cramer V = 0.447
Permanent (%)	45.90	73.90	-	75.70	-	-
Fixed term (%)	50.40	17.00	-	7.80	-	-
Other (%)	3.80	9.20	-	16.50	-	-
**Job type**	-	-	-	-	$\chi_{\left(2\right)}^{2}$ = 79.71***	Cramer V = 0.224
Full-time employee (%)	94.90	78.80	-	93.80	-	-
Part-time employee (%)	5.10	21.20	-	6.20	-	-
**How many hours do you work in a typical week? (M; s.d.)**	40.76^b^; 9.57	37.24^a^; 12.64	-	40.18^b^; 10.13	F_(2,1506)_ = 17.36***	Partial η^2^ = 0.021
**In a typical week, how many overtime (paid) hours do you work per week? (M; s.d.)**	4.99^b^; 5.07	3.29^a^; 4.13	-	3.43^a^; 6.93	F_(2,1506)_ = 15.78***	Partial η^2^ = 0.021
**Are you a supervisor?**	-	-	-	-	$\chi_{\left(2\right)}^{2}$ = 55.43***	Cramer V = 0.187
Yes (%)	52.00	28.80	-	38.60	-	-
No (%)	48.00	71.20	-	61.40	-	-
**Do you work in a group?**	-	-	-	-	$\chi_{\left(2\right)}^{2}$ = 112.88***	Cramer V = 0.268
Yes (%)	92.80	68.20	-	77.40	-	-
No (%)	7.20	31.80	-	22.60	-	-

Note: The ‘Other’ category under contract included the following options: Contingent/temporary work; Apprenticeship training; Paid internship; Other; and Do not have any contract of employment. Different superscripts (^a, b, c^) to different means indicate significant statistical differences, as highlighted by Tukey’s honestly significant difference (*hsd*) post hoc tests.

SA, South Africa; US, United States; s.d., standard deviation.

*** , *p* < 0.01;

* , *p* < 0.05.

### Instruments

*Cognitive Job Insecurity.* Cognitive job insecurity was assessed using the 9-item version of the Job Security Index (JSI, Probst, 2003). This scale was developed in order to evaluate ‘the perceived stability and continuance of one’s job as one knows it’ (p. 452). Participants rated a list of adjectives and phrases concerned with the future of their job using a 3-point scale (yes = 3, don’t know = 2, no = 0). These response options were modelled after the Job Descriptive Index since prior research (e.g. Hanisch, 1992) indicated this format allows for respondents with even very low reading ability to comprehend and discriminate among the categories; additionally, the asymmetrical 3/2/0 scoring is based on analyses by Hanisch (1992) that indicate the ‘don’t know’ response is not a neutral response, but rather is psychometrically closer to a ‘yes’ (i.e. higher insecurity) response than a ‘no’ response. Four items were negatively worded (e.g. ‘Unpredictable’, ‘Up in the air’), while five were positively worded (e.g. ‘Stable’, ‘My job is almost guaranteed’), and the order of presentation was mixed within the scale in order to avoid potential response biases. Items were recoded as necessary such that higher scores reflect greater cognitive job insecurity.

### Affective job insecurity

Affective job insecurity was assessed using the 9-item version of the Job Security Survey (JSS, Probst, 2003). This scale was developed in order to capture the ‘evaluative responses one might have to a perceived level of job security’ (p. 455). Participants rated a list of adjectives and phrases concerned with the stability of their job using the same response format of the JSI. Four items were negatively worded (e.g. ‘Upsetting how little job security I have’, ‘Unacceptably low’), while five were positively worded (e.g. ‘Looks optimistic’, ‘Never been more secure’). As with the JSI items, the order of presentation of both positive and negative JSS items was balanced within the scale, and items were recoded as needed such that higher scores reflect greater affective job insecurity.

### Procedure

A convenience sampling method was employed for both the SA and Italian data collection, and no incentives were offered to participants in these two countries. Online data collection was used in the US, China and SA, while a paper-based survey was distributed in Italy. In the US, participants were recruited with an online human subjects’ crowdsourcing platform (i.e. Amazon Mechanical Turk) as part of a larger research project on the antecedents, moderators and outcomes of job insecurity. Only individuals with an established track record of providing high quality data to previous crowd-sourced tasks (i.e. ‘high reputation’ participants; see Peer et al., 2014) were recruited. In addition, to circumvent any potential self-selection, based on potential participants’ existing perceptions of the constructs being measured, it was only indicated that participants needed to be currently employed and would be responding to a survey about their ‘work environment’. Upon completion of the survey, a small incentive ($2.00) was offered for participation. A similar strategy was followed in China by recruiting employees of Chinese enterprises through a well-known online survey platform (sojiang.com) in China. Respondents received a small reward ($2.83) for taking part in the survey.

In all the samples, the language of administration was the official national language of the country (i.e. English in the United States, Chinese in China and Italian in Italy). Because of the multilingual environment in South Africa (11 official national languages), only English and Afrikaans versions of the tests were administered to participants that indicated English or Afrikaans as their first (i.e. home) language. Translation and back translation procedures (Behling & Law, 2000) were utilised to create Afrikaans, Chinese and Italian versions of the JSI and JSS.

### Data analysis

**FIGURE 1:**
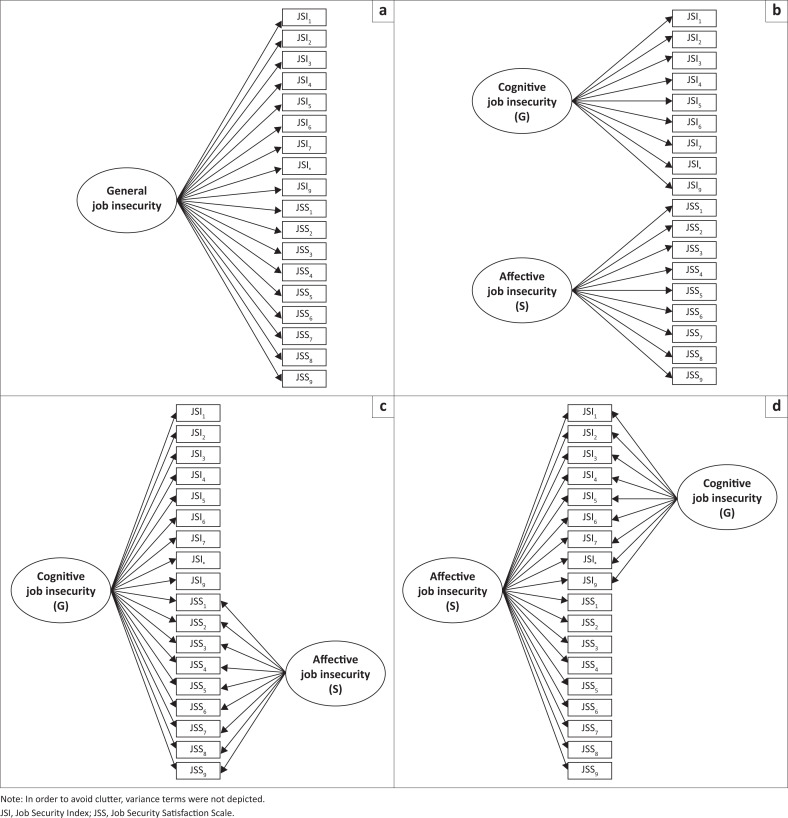
Conceptual bifactor-(*S*–1) model for the JSI and JSS Items. (a) Model 1 (M1) – Single factor, (b) Model 2 (M2) – Two oblique factors, (c) Model 3 (M3) – Bifactor-(*S*-1) structure (*Cognitive JI* as referent factor) and (d) Model 4 (M4) – Bifactor-(*S*-1) structure (*Affective JI* as referent factor).

A series of alternative factorial structures of the JSI and JSS measures were initially fitted separately for each country sample (see Figure 1). In line with Probst (2003), the first model posited a single factor underlining all JSI and JSS items (M1), while a second model posited two distinct latent and correlated common causes underlining, respectively, JSI and JSS items (M2). While in the first model, the single latent variable may be interpreted as a general dimension of job insecurity, in the second model, the posited latent variables are explicitly modelled to distinguish between CJI and AJI.

Following this, two further alternative factorial structures have been hypothesised and tested within the bifactor modelling approach (for an extensive review, see Reise, 2012). With this regard, it is important to note that the great majority of bifactor models proposed in the literature consisted in fully symmetrical bifactor structures of psychometric measures (Eid et al., 2017). In such models, all indicators load onto a general (G) factor and there are as many specific factors (S) as many specific constructs or facets are intended to be modelled, and both G and S factors are specified as orthogonal. As recently evidenced by Eid et al. (2017), fully symmetrical bifactor models have several theoretical and empirical flaws.

Firstly, S factors should be specified to model all specific constructs and facets under investigation only if they are structurally interchangeable. Specifically, two S factors may be considered as structurally interchangeable when they are randomly drawn from their universe of units (e.g. method factors associated with two randomly selected colleagues evaluating one’s job performance). In our case, CJI and AJI are clearly non-structurally interchangeable, as they represent two constructs explicitly proposed to assess different aspects of the broader concept of job insecurity (Cheng & Chan, 2008; Jiang & Lavaysse, 2018).

Secondly, fully symmetrical bifactor models generally produce estimates at odds with researchers’ expectations, such as issues with model convergence, negative variances of S factors and/or unexpected non-significant (even negative) factor loadings on the S factors (Eid et al., 2017; Heinrich et al., 2020), which are all typical signs of empirical non-identification of the estimated model (Bollen, 1989).

Thirdly, a limitation associated with fully symmetrical bifactor models is that the interpretation of both G and S factors is unclear (Bonifay et al., 2017), as the items are not clearly defined in terms of conditional expectations with respect to both general and specific latent variables (Eid et al., 2017). In order to overcome this limitation, Eid et al. (2017) proposed a modified version of the typical bifactor factorial structure, namely the bifactor-(*S*-1) model. Unlike other models, the bifactor-(*S*-1) factorial structure posits a reference general (G) factor (as for fully symmetrical bifactor models), and a number of specific factors (S) equal to that of the facets to be modelled minus one (for recent applications, see Burns et al., 2019; Heinrich et al., 2020). In this case, G and S factors are theoretically and mathematically defined as orthogonal, but S factors are allowed to covary. In this sense, the choice of the reference domain is crucial, because different options lead to different interpretation of both G and S factors and can alter the final estimates and overall fit of the model (Heinrich et al., 2020, 2021). The interpretation of both G and S factors in bifactor-(*S*-1) model is well-defined and straightforward. Specifically, the G factor represents the domain for which no S factor has been specified, while the interpretation of S factors is conditional on G: they represent residual latent constructs reflecting that part of the shared variance between the items which is independent from the referent domain.

This methodological framework allowed us to formulate the third and the fourth alternative models (i.e. M3 and M4) of our set. In M3, CJI was specified as the referent domain (G), while AJI represents the S factor, and this formalisation of referent and specific domains was inverted in M4. Although M3 reflects the most theoretically sound model (Jiang & Lavaysse, 2018; Lazarus & Folkman, 1984; Weiss & Cropanzano, 1996), M4 also represents a plausible option.

As the rating scale of JSI and JSS measures was on a three-option format, items were treated as ordered categorical variables (Flora & Curran, 2004). Thus, all models were tested using the least square mean and variance adjusted (WLSMV) estimators (Muthén & Muthén, 1998–2018) and a pairwise deletion strategy to handle missing data. Overall model fit was evaluated using multiple indices (Hu & Bentler, 1999a; 1999b; Kline, 2015): (1) WLSMVχ^2^ test statistic; (2) root mean square error of approximation (RMSEA); (3) Comparative Fit Index and Tucker–Lewis Fit Indices (respectively, CFI and TLI) and (4) standardised root mean square residual (SRMR). As not all models were nested (e.g. M3 and M4 were not nested within M2), the best-fitting model was determined by comparing different information criteria within each country sample. Specifically, Akaike’s Information Criterion, AIC (1973), the Bayesian Information Criterion (BIC, Schwarz, 1978) and the sample-size adjusted BIC (ABIC, Sclove, 1987) were computed by using appropriate formulas adapted from Yamaoka et al. (1978) based on the minimum value of the fitting function of the WLSMV estimators. Specifically, the two candidate models displaying lower values from AIC, BIC and ABIC indices were further compared. A ∆AIC > 10 indicates considerably less support for the model with the highest AIC (Burnham & Anderson, 2004), as well as both ∆BIC and ∆ABIC > 6 provide strong evidence for rejecting the model with higher values in these information criteria (if > 10 such evidence may be interpreted as very strong, see Kass & Raftery, 1995).

Once the most appropriate model was established for each country sample, cross-cultural measurement invariance of JSI and JSS measures was tested using a build-up strategy (Millsap & Yun-Tein, 2004). This procedure consists of testing a series of hierarchically nested models (i.e. configural, metric, scalar and strict invariance models) and comparing their fit in order to evaluate if different psychometric properties of the measures under investigation can be generalised across samples. For this purpose, we used the THETA parameterisation approach of Mplus 8.4 (Muthén & Muthén, 1998–2018) and all latent variables were scaled by fixing their first factor loading to unity.

Given that the ∆WLSMVχ^2^ test statistic is typically largely inflated by Type I error (see Sass et al., 2014), statistical comparison between adjacent nested models was carried out by evaluating the differences between values of their alternative fit indices (i.e. ∆RMSEA, ∆CFI and ∆TLI). Moreover, as many cut-offs have been proposed in the literature to establish different levels of invariance when using WLSMV estimators (for an overview, see Sass et al., 2014; Svetina et al., 2020), we set the rejection cut-off criteria to the most conservative available values: ∆RMSEA ≤ –0.007 (Meade et al., 2008), ∆CFI ≤ –0.002 (Svetina & Rutkowski, 2017) and ∆TLI < –0.001 (Marsh et al., 2010). If the full set of equality constraints specified in a given model is not tenable, partial invariance can still be pursued (Byrne et al., 1989; Vandenberg & Lance, 2000). In this case, modification indices were inspected, and equality constraints were released one by one (Millsap & Yun-Tein, 2004) until the most restrictive model did not significantly differ in terms of model fit with respect to the less restrictive one. In line with recent simulation studies (Lai et al., 2021), we established that the minimum level to achieve partial cross-cultural measurement invariance of the study measures was no more than one-third of noninvariant parameters over the total.

### Ethical considerations

In the United States, Italy and China, because of the anonymity of the data and low risk to participants, the respective Institutional Review Boards classified the studies as exempt. The South African study was classified as low risk because of anonymity of the respondents, but a full review by the relevant higher educational institution’s research ethics committee was still required before clearances were obtained. Ethical clearance to conduct the study was obtained from the Research Ethics Committee: Social, Behavioural and Education Research at Stellenbosch University in the Western Cape (Project ID#8713). This included the ethics clearances granted by other international institutions.

Participants indicated their consent with an online consent statement. The anonymity of reported responses, as well as the secure password protection of data, was communicated to participants.

## Results

Proportion of responses in answer categories for both JSI and JSS items is provided in Online Appendix 1 (Table S2). Table 2 shows fit indices of the alternative models tested separately per each country sample. As can be noted from the inspection of information criteria values, the two best-fitting models were M3 and M4 for the Chinese, Italian and SA samples, while in the case of US data were M2 and M3. In the case of Italian, SA and US data, the M3 model showed consistently lower values of all information criteria indices than for the second best fitting model (albeit in the US data, the ∆BIC between M2 and M3 was only slightly lower than 1). For the Chinese data, M4 seems to outperform M3 when ∆AIC and ∆ABIC are inspected, while ∆BIC showed the opposite pattern. However, estimates provided by M4 highlighted some unexpected results: specifically, two items of the JSS measure loaded negatively on the specific factor, as well as the variance term of the S factor was not statistically significant, suggesting that the residual CJI factor is substantially meaningless in that sample.

**TABLE 2:** Overall fit indices of the alternative factorial models for each country sample.

Model	WLSMVχ^2^	*df*	*p*	RMSEA	90% CI	CFI	TLI	SRMR	AIC	BIC	ABIC
Chinese sample (*N* = 629)											
M1 – Single factor	608.983	135	< 0.001	0.075	0.069 – 0.081	0.969	0.965	0.058	28.655,787	28.895,770	28.724,327
M2 – Two oblique factors	518.603	134	< 0.001	0.068	0.061 – 0.074	0.975	0.972	0.052	28.529,696	28.774,123	28.599,505
M3 – Bifactor-(*S*-1), *CJI* as referent	513.222	126	< 0.001	0.070	0.064 – 0.076	0.975	0.972	0.051	28.493,614	28.529,696	28.773,594
M4 – Bifactor-(*S*-1), *AJI* as referent	487.623	126	< 0.001	0.068	0.061 – 0.074	0.977	0.972	0.051	28.455,028	28.735,008	28.534,991
Italian sample (*N* = 482)			< 0.001								
M1 – Single factor	1,065.277	135	< 0.001	0.120	0.113 – 0.126	0.956	0.950	0.096	25.338,036	25.563,645	25.392,254
M2 – Two oblique factors	772.084	134	< 0.001	0.099	0.093 – 0.106	0.970	0.965	0.079	24.968,425	25.198,212	25.023,647
M3 – Bifactor-(*S*-1), *CJI* as referent	411.192	126	< 0.001	0.069	0.061 – 0.076	0.986	0.984	0.051	24.815,539	25.078,749	24.878,793
M4 - Bifactor-(*S*-1), *AJI* as referent	789.355	126	< 0.001	0.105	0.098 – 0.112	0.969	0.962	0.078	24.913,832	25.177,043	24.977,086
SA sample (*N* = 345)			< 0.001								
M1 – Single factor	532.587	135	< 0.001	0.092	0.084 – 0.101	0.989	0.988	0.041	15.714,388	15.921,940	15.750,637
M2 – Two oblique factors	438.306	134	< 0.001	0.081	0.073 – 0.090	0.992	0.990	0.033	15.507,055	15.718,450	15.543,975
M3 – Bifactor-(*S*-1), *CJI* as referent	357.591	126	< 0.001	0.073	0.064 – 0.081	0.994	0.992	0.028	15.385,218	15.627,361	15.427,508
M4 – Bifactor-(*S*-1), *AJI* as referent	439.691	126	< 0.001	0.085	0.076 – 0.094	0.991	0.990	0.033	15.493,850	15.735,993	15.536,140
US sample (*N* = 486)			< 0.001								
M1 – Single factor	815.187	135	< 0.001	0.102	0.095 – 0.109	0.977	0.973	0.055	21.886,196	22.112,251	21.940,858
M2 – Two oblique factors	581.035	134	< 0.001	0.083	0.076 – 0.090	0.985	0.982	0.039	21.444,425	21.674,667	21.500,100
M3 – Bifactor-(*S*-1), *CJI* as referent	513.788	126	< 0.001	0.080	0.072 – 0.087	0.987	0.984	0.033	21.410,007	21.673,738	21.473,779
M4 – Bifactor-(*S*-1), *AJI* as referent	596.312	126	< 0.001	0.088	0.081 – 0.095	0.984	0.980	0.039	21.453,078	21.716,809	21.516,850

CJI, cognitive job insecurity; AJI, affective job insecurity; WLSMVχ^2^, Chi-square based on weighted least squares estimators with means and variances adjusted; *df*, degrees of freedom; RMSEA, root mean square error of approximation; 90% CI, 90% confidence interval; CFI, comparative fit index; TLI, Tucker–Lewis fit index; SRMR, standardised root mean squared residual; AIC, Akaike information criterion; BIC, Bayesian information criterion; ABIC, sample-size adjusted BIC; SA, South Africa; US, United States.

Given these results, M3 was retained as the final model for all country samples. As can be noted from Table 2, fit indices of the M3 model ranged from acceptable (RMSEA) to good (CFI and TLI) in all country samples. Consistent with the bifactor-(*S*-1) modelling approach, the interpretation of G and S factors specified in M3 is clear and theoretically well-defined. On the one hand, the G factor represents the referent domain of the JSI and JSS measures, reflecting the cognitive component associated with job insecurity perceptions underlining the entire set of items. On the other hand, the S factor reflects the affective (evaluative) component of job insecurity which is independent from the referent (cognitive) domain. More specifically, the latent score on the S factor can be interpreted as the degree to which an individual activates higher (or lower) affective evaluative reactions to job insecurity compared to other individuals having the same latent score on the G (cognitive) factor.

Table 3 shows results of the measurement invariance tests of M3 across the country samples. As can be noted, full metric invariance was not supported by the data (for the full pattern of fixed, invariant and non-invariant parameters, see also Table S3 of Online Appendix 1). Specifically, modification indices progressively suggested to release two-factor loadings on the G factor in the Chinese sample (for JSI_7_ and JSI_9_ items), four in the case of the Italian sample (for JSI_5_, JSI_6_, JSI_8_, and JSS_6_ items) and one in the case of both SA and US samples (for JSS_6_). Moreover, a single factor loading on the S factor was released in the Italian sample (for JSS_8_). After releasing these constraints, the partial metric invariance was reached. Thus, we tested the partial metric full scalar model. Also, in this case, some constraints on item thresholds led to a significant worsening in model fit. Specifically, ten thresholds were released in the Chinese sample, nine in the Italian sample, six in the SA sample and five in the US sample (see Table S3 of Online Appendix 1). After releasing these constraints, the partial metric scalar model was tenable. (As the residual variance of JSS_7_ was no longer significant for the Italian sample, this parameter was fixed to 0 for that group [Bollen, 1989]). Finally, we imposed equalities on items’ residual variances across the country samples. A single equality constraint was released in the Chinese, Italian and SA samples (for JSS_3_, JSS_2_ and JSS_6_ items, respectively), while there was no need to release any additional constraint on the US sample. Overall, we reached the partial metric, partial scalar, partial strict invariance of our substantive bifactor-(*S*-1) structure. As can be noted from Table 4, the proportion of non-invariant parameters was consistently low for all country samples. This evidence supported the cross-cultural generalisability of the final factorial structure of JSI and JSS items, highlighting very similar psychometric properties across the country samples involved in the present study. Moreover, as the noninvariant parameters were lower than one-third (see Lai et al., 2021), we can conclude that the measurement properties of the study scales were substantially generalisable across countries.

**TABLE 3:** Measurement invariance of the final bifactor-(*S*-1) Model (M3).

Levels of measurement invariance	WLSMVχ^2^	*df*	*p*	RMSEA	90% CI	CFI	TLI	SRMR	MC	∆RMSEA	∆CFI	∆TLI
1. Configural invariance	1,793.333	504	< 0.001	0.073	0.069–0.076	0.988	0.985	0.044	-	-	-	-
2. Metric invariance	2,641.371	579	< 0.001	0.086	0.082–0.089	0.980	0.979	0.082	2 vs. 1	−0.008	−0.008	−0.006
2a. Partial metric invariance	1,994.173	570	< 0.001	0.072	0.068–0.075	0.987	0.986	0.029	2a vs. 1	−0.001	−0.001	0.001
3. Scalar invariance	3,137.500	618	< 0.001	0.092	0.088–0.095	0.976	0.976	0.053	3 vs. 2a	−0.011	−0.008	−0.010
3a. Partial scalar invariance	2,095.291	598	< 0.001	0.072	0.068–0.075	0.986	0.986	0.048	3a vs. 2a	−0.001	−0.001	0.000
4. Strict invariance	2,384.292	621	< 0.001	0.076	0.073–0.080	0.983	0.984	0.060	4 vs. 3a	−0.003	−0.003	−0.002
4a. Partial strict invariance	2,129.029	618	< 0.001	0.071	0.068–0.074	0.986	0.986	0.052	4a vs. 3a	0.000	0.000	0.000

WLSMV χ^2^, Chi-square based on weighted least squares estimators with means and variances adjusted; *df*, degrees of freedom; RMSEA, root mean square error of approximation (90% confidence interval); CFI, Comparative Fit Index; TLI, Tucker–Lewis Fit Index; SRMR, standardised root mean squared residual; MC, model comparison.

**TABLE 4:** Number of non-invariant parameters from the most restrictive measurement invariance model (4a).

Samples	Factor loadings onto the G Factor (*n* = 17)	Factor loadings onto the S Factor (*n* = 8)	Thresholds (*n* = 36)	Residual variances (*n* = 18)	Total non-invariant parameters (*n* = 79)	%
Chinese sample (*N* = 629)	2	-	10	1	13	16.5
Italian sample (*N* = 482)	4	1	9	1	15	19.0
SA sample (*N* = 345)	1	-	6	1	7	8.9
US sample (*N* = 486)	1	-	5	-	6	7.6

Note: In the bottom line, values in parentheses refer to the total number of parameters estimable for each country sample. In the last column, values in parentheses refer to the proportion of non-invariant parameters.

SA, South Africa; US, United States

Finally, we calculated consistency (*Con*), specificity (*Spec*) and reliability (*Rel*) coefficients (Eid et al., 2017; Heinrich et al., 2020) for each item from the most restrictive measurement invariance model (Table 5). While consistency coefficients reflect the proportion of the true score of each item attributable to individual differences in the CJI referent domain (G), specificity coefficients reflect to what extent such true score is accounted for by the AJI residual factor (S) after controlling for G. *Rel* coefficients represent the proportion of the observed total score of the items which is accounted for by error-free individual differences on both G and S factors. As can be noted, the G factor accounts for the largest proportion of items’ true scores in all country samples (average *Con* coefficients ranged from 0.721 to 0.884), but the S factor represented a significant unique source of true score variability in all cases (average *Spec* coefficients ranged from 0.107 to 0.279). Finally, a very high proportion of items’ observed scores was accounted for by reliable individual differences attributable to latent factors (average *Rel* coefficients ranged between 0.650 and 0.863 across country samples). Overall, we can conclude that CJI represents a strong common referent domain underlining both JSI and JSS items, but the AJI residual factor provided unique and valuable added information regarding the shared variability among JSS items in all country samples.

**TABLE 5:** Estimates from the final multi-group model (4a) and consistency, specificity and reliability coefficients.

Items	Chinese sample (*n* = 629)					*p*	Italian sample (*n* = 482)					*p*	SA sample (*n* = 345)					*p*	US sample (*n* = 486)					*p*
	λ_(G)_	λ_(S)_	*Con*	*Spec*	*Rel*		λ_(G)_	λ_(S)_	*Con*	*Spec*	*Rel*		λ_(G)_	λ_(S)_	*Con*	*Spec*	*Rel*		λ_(G)_	λ_(S)_	*Con*	*Spec*	*Rel*	
JSI_1_	0.873	-	1.000	0.000	0.762	-	0.945	-	1.000	0.000	0.893	-	0.957	-	1.000	0.000	0.916	-	0.918	-	1.000	0.000	0.843	-
JSI_2_	0.830	-	1.000	0.000	0.689	-	0.940	-	1.000	0.000	0.884	-	0.933	-	1.000	0.000	0.870	-	0.937	-	1.000	0.000	0.878	-
JSI_3_	0.809	-	1.000	0.000	0.654	-	0.843	-	1.000	0.000	0.711	-	0.898	-	1.000	0.000	0.806	-	0.926	-	1.000	0.000	0.857	-
JSI_4_	0.873	-	1.000	0.000	0.762	-	0.713	-	1.000	0.000	0.508	-	0.940	-	1.000	0.000	0.884	-	0.943	-	1.000	0.000	0.889	-
JSI_5_	0.805	-	1.000	0.000	0.648	-	0.955	-	1.000	0.000	0.912	-	0.934	-	1.000	0.000	0.872	-	0.942	-	1.000	0.000	0.887	-
JSI_6_	0.833	-	1.000	0.000	0.694	-	0.860	-	1.000	0.000	0.740	-	0.936	-	1.000	0.000	0.876	-	0.911	-	1.000	0.000	0.830	-
JSI_7_	0.860	-	1.000	0.000	0.740	-	0.842	-	1.000	0.000	0.709	-	0.935	-	1.000	0.000	0.874	-	0.901	-	1.000	0.000	0.812	-
JSI_8_	0.796	-	1.000	0.000	0.634	-	0.916	-	1.000	0.000	0.839	-	0.838	-	1.000	0.000	0.702	-	0.797	-	1.000	0.000	0.635	-
JSI_9_	0.762	-	1.000	0.000	0.581	-	0.804	-	1.000	0.000	0.646	-	0.958	-	1.000	0.000	0.918	-	0.938	-	1.000	0.000	0.880	-
JSS_1_	0.836	0.120	0.980	0.020	0.713	-	0.705	0.191	0.932	0.068	0.534	-	0.828	0.141	0.972	0.028	0.705	-	0.835	0.189	0.951	0.049	0.733	-
JSS_2_	0.739	0.428	0.749	0.251	0.729	-	0.448	0.491	0.454	0.546	0.442	-	0.798	0.549	0.679	0.321	0.938	-	0.705	0.642	0.547	0.453	0.909	-
JSS_3_	0.844	0.222	0.935	0.065	0.762	-	0.773	0.384	0.802	0.198	0.745	-	0.882	0.275	0.911	0.089	0.854	-	0.809	0.334	0.854	0.146	0.766	-
JSS_4_	0.667	0.248	0.879	0.121	0.506	-	0.923	0.272	0.920	0.080	0.926	-	0.842	0.372	0.837	0.163	0.847	-	0.756	0.443	0.744	0.256	0.768	-
JSS_5_	0.775	0.211	0.931	0.069	0.645	-	0.811	0.416	0.792	0.208	0.831	-	0.906	0.292	0.906	0.094	0.906	-	0.845	0.361	0.846	0.154	0.844	-
JSS_6_	0.660	0.326	0.804	0.196	0.542	-	0.479	0.710	0.313	0.687	0.734	-	0.941	0.125	0.983	0.017	0.901	-	0.904	0.333	0.881	0.119	0.928	-
JSS_7_	0.549	0.288	0.784	0.216	0.384	-	0.775	0.631	0.601	0.399	0.999	-	0.808	0.414	0.792	0.208	0.824	-	0.713	0.484	0.685	0.315	0.743	-
JSS_8_	0.799	0.342	0.845	0.155	0.755	-	0.495	0.845	0.255	0.745	0.959	-	0.866	0.441	0.794	0.206	0.944	-	0.792	0.533	0.688	0.312	0.911	-
JSS_9_	0.673	0.233	0.893	0.107	0.507	-	0.789	0.422	0.778	0.222	0.801	-	0.892	0.301	0.898	0.102	0.886	-	0.825	0.368	0.834	0.166	0.816	-
Average	0.777	0.269	0.893	0.107	0.650	-	0.779	0.485	0.721	0.279	0.767	-	0.894	0.323	0.884	0.116	0.863	-	0.855	0.410	0.813	0.187	0.829	-
s.d.	0.086	0.084	0.509	0.491	0.105	-	0.155	0.198	0.379	0.621	0.159	-	0.051	0.130	0.134	0.866	0.068	-	0.078	0.125	0.280	0.720	0.076	-
Var_(G)_						3.199***						7.294***						16.482***						8.702***
Var_(S)_						0.050***						0.409***						0.366***						0.338***

λ_(G)_, standardised loading on the referent factor; λ_(S)_, standardised loading on the specific factor; *Con*, consistency coefficient (proportion of true score because of the referent factor); *Spec*, specificity coefficient (proportion of true score because of the specific factor); *Rel*, proportion of true score over the total score; Var_(G)_, estimated variance of the referent factor; Var_(S)_, estimated variance of the specific factor; s.d., standard deviation.

*** , All factor loadings were statistically significant for *p* < 0.01

### Discussion

#### Theoretical contribution and practical measurement implications

Consistent and reliable cross-study and country comparisons on job insecurity hinge on assessments of measurement invariance in this domain (e.g. Vander Elst et al., 2014). The purpose of this study was to investigate the measurement invariance of the JSI and JSS over four samples, each derived from one country on four different continents (the US, Italy, China and SA). Partial metric, partial scalar and partial strict invariance for a bifactor-(*S*-1) model (M3) were achieved, rendering meaningful cross-national group comparisons permissible for this model. The results make several contributions to the current job insecurity literature.

Firstly, a series of competing models were tested. Overall, these represented a unique approach to investigating the cognitive–affective job insecurity relationship. Although the cognitive–affective distinction is well supported (e.g. Jiang & Probst, 2014; Probst, 2003, 2008; Reisel & Banai, 2002), the distinct nature of their interrelationship is less often reported. While Eid et al. (2017, p. 555) state that the bifactor- (*S*-1) model is mainly applicable when a ‘clear candidate for a reference domain’ exists, we argued in favour of testing competing models, given that no clear theoretical justification for either model existed from previous studies. The results revealed that the consistently best fitting model (i.e. Model 3) represented affective job insecurity as conditional to cognitive job insecurity, providing additional support for the distinctiveness of these two constructs and clarification of the complex relationship between them (e.g. Jiang & Lavaysse, 2018).

Theoretically, this result suggests that in this cross-national dataset, a model where cognitive job insecurity is specified as the reference domain outperforms a model where affective job insecurity is assigned this status. Practically, this suggests that interpretations of affective job insecurity scores hinge upon levels of cognitive job insecurity. Moreover, it suggests that across all samples, the nature of job insecurity is best demarcated as affective job insecurity being conditional to cognitive job insecurity, suggesting that interpretations of affective job insecurity rely on levels of cognitive job insecurity.

Theoretically, this result also aligns with both the cognitive appraisal (Lazarus & Folkman, 1984) and affective events theories (Weiss & Cropanzano, 1996), when applied to the job insecurity domain. Moreover, it suggests that this theoretical interpretation may replicate over different cultural–cross-national contexts. That is, the potential threat of job loss, i.e. cognitive job insecurity, as suggested by cognitive appraisal theory, could with confidence be assigned the status of *primary* appraisal, the process through which the meaning and significance of an event (i.e. potential job loss) is recognised (Lazarus, 1991). Affective job insecurity, hereafter, represents a *secondary* appraisal (Smith & Pope, 1992) contingent on coping resources envisioned to mitigate the severity of the threat inherent to the job loss appraisal. Similarly, as predicted by affective events theory (Weiss & Cropanzano, 1996), emotional or affective reactions (such as anxiety or worry) stem from the cognitive appraisal of events (evaluated for relevance to well-being) as proximal causes. Recently, Charkhabi (2019, p.2) argued that ‘actual job insecurity and appraisal of job insecurity, are two distinct constructs’ and showed that a hindrance appraisal of job insecurity mediates the relationship between quantitative job insecurity and emotional exhaustion. Moreover, strong evidence of the mediator effect of AJI in the relationships between CJI and a broad spectrum of workplace outcomes (see Jiang & Lavaysse, 2018) further underscores this notion.

This study provides strong cross-national evidence of the notion that ‘AJI can be considered as an emotional reaction to CJI’ (Jiang & Lavaysse, 2018, p. 2316). It, furthermore, underscores the notion that disentangling AJI and CJI in studies on JI may provide a stronger theoretical approach to understand the psychological mechanism driving the outcomes of JI. For example, a recent longitudinal study (Griep et al., 2021) revealed that perceptions of job insecurity influence mental health complaints when persistent job insecurity was present. However, the JI measure utilised in this study (Vander Elst et al., 2014) appears to reflect CJI only, and therefore perhaps missed the additional benefits offered by recognising that CJI and AJI as two separate appraisals with unique explanatory power in the stressor appraisal process.

The measurement invariance results indicated that partial metric, partial scalar and partial strict invariance for the substantive bifactor-(*S*-1) Model 3 emerged, conditional on certain parameters being modified between groups, suggesting specific conclusions regarding the translated versions of the JSI and JSS. More specifically, sufficient evidence emerged supporting the factorial structure of Model 3 (i.e. configural invariance model) over the four samples. This implies that the manifest measures induced similar conceptual frames of reference in each of the groups (Riordan & Vandenberg, 1994; Vandenberg & Lance, 2000).

The partial metric invariance results suggest similarity in the scaling units across countries, implying that meaningful interpretation of item scores across countries are permissible. That is, the majority of the translated JIS and JSS item observed scores are similarly calibrated to the two job insecurity factor scores across countries, given the unique relationship posited between them in the bifactor-(*S*-1) model. Moreover, the results revealed evidence of partial strict invariance (i.e. error variance invariance), implying invariance of the measurement errors of the translated JIS and JSS versions, and a partial lack of measurement bias. In conclusion, these results suggest that the Italian, Chinese, English and Afrikaans versions of the cognitive and affective job insecurity measures are invariant, a permissible conclusion as at least partial invariance of the parameters was found (see Vandenberg & Lance, 2000; Milfont & Fischer, 2010). This evidence is further supported by recent simulation studies (Lai et al., 2021), suggesting that less than one-third of noninvariant parameters do not affect the overall validity of a psychometrically sound measure.

This study extends measurement invariance research of job insecurity in several ways. Firstly, it represents the initial attempt (to the knowledge of the authors) to conduct invariance analysis on the two forms of job insecurity, reflecting their distinctiveness, but also significant interrelatedness. The only other study of this nature was conducted on a unidimensional global measure of job insecurity (i.e. Vander Elst et al., 2014). Secondly, the asymmetrical rating scale employed by the JSI and JSS is based on prior research by Hanisch (1992) indicating that a ‘don’t know’ response is psychometrically closer to a negative response (i.e. reflecting greater job insecurity) rather than being equidistant between the negative and positive response options. Because simulation studies (e.g. Bovaird & Koziol, 2012) indicate that three-point response scale may not be treated as approximately continuous, this requires (and our study employed) suitable estimation techniques (i.e. weighted least squares estimators) to handle the resulting ordinal data. Thirdly, this study employed a bifactor-(*S*-1) model approach (Heinrich et al., 2020) to circumvent theoretical and empirical weaknesses present in fully symmetrical bifactor models (e.g. Eid et al., 2017) while extending this application in terms of its theoretical contribution on the cognitive and affective job insecurity literature. Lastly, this study attempts to answer the call for measurement invariance studies over a diverse set of language and cultural groups (Bazzoli & Probst, 2023) on the JSI and JSS. It enhances confidence in the use of these measures, also in the African context, where measurement equivalence should be a particularly pressing issue, given the diversity of multilingual and cultural groups on which Western developed (etic) measures are often applied.

### Limitations

Despite these contributions, some limitations must be acknowledged. Firstly, the cross-sectional nature of the data imposes some constraints on the conclusions derived from it. No information on the stability of the reliability and validity of the measures over time were included. Moreover, future studies should investigate directly potential sources of cross-national non-invariance of some parameters. A future cross-national longitudinal invariance study would strengthen the practical and theoretical implications of this research and could facilitate investigations into the differential prediction of job insecurity and work-related outcomes (e.g. job performance), cross-culturally. Secondly, we were limited in the comparisons that could be made regarding the composition of each country’s sample because of the kinds of demographic information that could be collected in each country. For example, different interpretations of job insecurity for temporary versus permanent employees, based on perceived psychological contract breach (e.g. De Cuiper & De Witte, 2006), may exist. Thirdly, no information about the representativeness of the samples for the respective countries is available. The South-African data, for example, only contain data for Afrikaans and English first-language respondents, thereby omitting a rather large portion of the population. Future research should attempt to use matched (i.e. on sociodemographic characteristics and type of job contracts) representative samples. Lastly, differences in administration (i.e. hard copy versus online) may have introduced administration bias, the effect of which may have resulted in method bias (Van de Vijver & Poortinga, 1997).
